# The digital divide in cardiovascular care: who gets left behind?

**DOI:** 10.1093/ehjdh/ztag011

**Published:** 2026-01-21

**Authors:** Joshua J Hon, Gerald Carr-White

**Affiliations:** Faculty of Medicine, Imperial College London, Exhibition Road, London SW7 2AZ, UK; Guy’s and St Thomas’ NHS Foundation Trust, Westminster Bridge Road, London SE1 7EH, UK

## Introduction

Digital transformation has become a defining inflection point in cardiovascular care, supported by robust evidence demonstrating measurable benefits across multiple domains of practice. Yet emerging evidence suggests that, despite the promise of these advances, we may inadvertently create new barriers for disadvantaged, high-risk patients who are likely to benefit most.

## Discussion

### Understanding the emerging disparities

Accumulating evidence reveals patterns of digital health disparities that cardiovascular practitioners must address. Patients aged 80 years and older demonstrate 76% reduced odds of accessing video telemedicine consultations compared with younger patients.^1^ Black patients in the USA have a 36% lower odds of utilizing video-based cardiovascular care, while those with limited English proficiency encounter 48% reduced access to digital health platforms.^2^ These differences suggest our current implementation approaches may unintentionally exclude vulnerable populations from technological advances. While it is important to acknowledge the challenges faced by these vulnerable populations, digital technology and artificial intelligence (AI) have been effectively utilized in certain contexts to address gaps in cardiovascular care for underserved patient populations, demonstrating that exclusion is not inevitable.

The geographical dimension proves equally concerning. Rural residents in high-income countries, who already face elevated cardiovascular mortality rates,^3^ may be further disadvantaged by video-based telemedicine consultations due to unreliable broadband access and limited digital literacy.^4^ The extent of disadvantage varies by specific digital health intervention, with robust evidence currently limited primarily to video telemedicine and remote patient monitoring platforms.^5,6^

### The unintended consequences of artificial intelligence implementation

Perhaps nowhere do we see greater potential for both benefit and unintended bias than in AI applications. As we develop cardiovascular AI systems, the datasets used for training may not fully represent the populations most burdened by disease. When algorithms are deployed at scale without comprehensive validation across diverse populations, we risk amplifying existing healthcare disparities.

Performance data reveal important patterns. Deep learning models for electrocardiogram interpretation show declining accuracy in patients over 80 years (area under the curve [AUC] 0.73 vs. 0.81 in younger adults),^7^ with heart failure prediction algorithms demonstrating AUC reductions from 0.80 in younger patients to 0.66 in older populations due to algorithmic bias.^8^

These findings highlight the importance of ensuring our AI systems perform equitably across all patient populations. When algorithms are trained predominantly on data from younger, more homogeneous populations, we may inadvertently create digital disparities mirroring historical healthcare inequities.

### The wearable technology challenge

Consumer wearables offer democratized cardiac monitoring, yet adoption data expose significant gaps. Despite cardiovascular disease affecting predominantly older adults, fewer than 20% of established cardiovascular patients utilize wearable devices, with consistent use reported by only half of adopters.^9^ While historical concerns have been raised regarding photoplethysmography-based pulse oximetry accuracy in individuals with darker skin tones,^10–13^ recent evidence suggests a more nuanced picture. The EquiOx study in critically ill patients found that although pulse oximetry measurements showed overall negative bias, the bias was actually less negative in darker skin categories,^14^ with newer devices demonstrating improved performance. Furthermore, limited evidence suggests that photoplethysmography-based heart rate measurement, which forms the foundation of most consumer wearables, does not appear to be significantly affected by skin tone.^15^ These findings highlight the importance of avoiding overgeneralizations, though continued vigilance regarding device performance across diverse individuals remains essential.

Even when wearables generate data, questions remain about clinical utility. Over 90% of AI-generated alerts from wearable devices prove clinically non-actionable, with atrial fibrillation diagnosed by consumer devices confirmed by cardiologists in only 34–65% of cases.^16^ This creates situations where some users receive excessive false alarms, contributing to anxiety,^17^ while others lack access to potentially beneficial monitoring.

### Reconsidering our digital strategy

The cardiovascular community is navigating digital transformation, recognizing both its transformative potential and imperatives it creates for equitable implementation.

Substantial infrastructure investments for comprehensive digital health equity—including broadband access, device subsidies, multilingual interfaces, and digital literacy training—represent significant costs requiring evaluation against alternative strategies for achieving digital health equity, including both technological modifications (such as simplified interfaces or offline-capable tools) and complementary non-digital interventions that address the same health outcomes in underserved populations.

Patient-facing digital health implementations—including consumer health applications, telemedicine platforms, and wearables—often assume patients possess smartphones, reliable internet access, and sufficient technological literacy. These assumptions may exclude elderly patients managing multiple conditions, immigrants navigating language barriers, and economically disadvantaged individuals managing competing priorities. These concerns primarily address consumer-facing technologies. However, provider-facing digital health applications, including AI-driven clinical support systems and electronic health record analytics, represent a substantial portion of digital health solutions with different equity implications that merit separate consideration.

### A comprehensive approach

The development of responsible AI and digital health approaches has been substantially advanced by foundational work from multiple organizations. The World Health Organization’s (WHO) Ethics and Governance of Artificial Intelligence for Health established six core principles—protecting human autonomy, promoting well-being and safety, ensuring transparency, fostering accountability, ensuring inclusiveness and equity, and promoting responsive and sustainable AI.^18^ The Guidelines International Network (GIN) has proposed eight principles specifically for AI use in the health guideline enterprise, including transparency, pre-planning, additionality, credibility, ethics, accountability, compliance, and evaluation.^19^ The National Academy of Medicine’s AI Code of Conduct provides a comprehensive framework addressing governance, development, and monitoring across the AI lifecycle.^20^ Building upon these established frameworks, cardiovascular-specific implementation requires tailored approaches.

Cardiovascular medicine can operationalize these established principles through specific actions. Training AI algorithms on diverse datasets to adequately represent high-risk populations addresses the inclusiveness principle while improving clinical validity. Designing interfaces that prioritize accessibility alongside functionality implements the equity and transparency principles in practical terms. Developing workflows that accommodate varying technological capabilities ensures the protection of human autonomy. Measuring success not only by adoption rates but by impact on health disparities across all populations enacts the accountability and evaluation principles.

The evaluation paradigm for cardiovascular digital health tools should extend beyond technical validation metrics. Following the principles established by the WHO and echoed in the GIN guidance, assessment frameworks should include algorithmic performance across demographic strata, accessibility of implementation requirements, transparent reporting of training data composition, mechanisms for ongoing monitoring and refinement, and measurable impact on health disparities.

### Towards equitable digital innovation

Digital health systems may reflect their creators’ perspectives and priorities when development teams lack diversity or when insufficient attention is paid to potential biases. However, this limitation is not universal, and thoughtfully designed development processes that prioritize diverse input and rigorous bias testing can substantially mitigate these concerns.

Cardiovascular medicine has an opportunity to ensure technological advancement serves equity through thoughtful resource allocation and honest examinations of potential biases. Applying the principles outlined above, we can reconsider how to conceive, develop, and successfully implement digital health technologies in cardiovascular care. The potential benefits are substantial: ensuring that our most powerful tools reach those most vulnerable to cardiovascular disease.

### Conclusion

The digital transformation of cardiovascular care presents remarkable opportunities and important challenges requiring thoughtful navigation. As we develop increasingly sophisticated technologies, we can ensure innovation serves all patients equitably, particularly those high-risk populations who stand to benefit most but may face access barriers. The fundamental question is not whether we can develop sophisticated AI algorithms or capable wearable devices, but whether we can implement these tools to reduce rather than exacerbate cardiovascular health disparities. With thoughtful design, inclusive development, and equity-focused metrics, digital health technologies could advance cardiovascular health equity. Our choices will determine whether digital transformation becomes a force for greater health equity or an inadvertent contributor to widening disparities.
